# Incremental burden of relapse in patients with major depressive disorder: a real-world, retrospective cohort study using claims data

**DOI:** 10.1186/s12888-022-03793-7

**Published:** 2022-03-01

**Authors:** Maëlys Touya, Debra F. Lawrence, Anne Kangethe, Lambros Chrones, Themmi Evangelatos, Michael Polson

**Affiliations:** 1 Lundbeck LLC, 6 Parkway North, Deerfield, IL 60015 USA; 2 Takeda Pharmaceuticals U.S.A., Inc, Lexington, MA USA; 3 Magellan Rx Management, Scottsdale, AZ USA

**Keywords:** Major depressive disorder, Relapse, Burden, Real-world data, Retrospective study, Claims data, Cost, Health care utilization, Adherence, Antidepressants

## Abstract

**Background:**

Relapse is common in major depressive disorder (MDD). In this study, we evaluated the incremental health care burden of relapse in patients with MDD.

**Methods:**

This real-world retrospective cohort study used administrative medical and pharmacy claims data to identify commercially insured adult patients in the United States diagnosed with MDD who initiated a new antidepressant between January 1, 2012, and September 30, 2017. All-cause health care resource utilization, total costs, and medication adherence were evaluated in two cohorts: patients with and patients without relapse. Relapse was defined as suicide attempts, psychiatric hospitalization, mental health–related emergency department (ED) visit, use of electroconvulsive therapy, or reinitiation of treatment after a gap ≥6 months.

**Results:**

The study population included 14,186 patients (7093 baseline-matched patients per cohort). The mean follow-up period was 27.5 and 26.0 months for patients with and patients without relapse, respectively. Patients with relapse had significantly higher rates of hospitalization (16.6% vs 8.5%; *p <* .0001) and ED visits (54.8% vs 34.7%; *p <* .0001) than patients without relapse. The total costs for patients with relapse were significantly higher ($12,594 vs $10,445;  *p <* .0001). Patients with relapse were also less adherent to antidepressants (mean proportion of days covered, 0.43 vs 0.49; *p <* .0001).

**Conclusions:**

Relapse of MDD was associated with increased total costs and health care utilization and lower adherence to antidepressants. Reducing the risk of relapse may result in a reduction of the associated health care burden; however, findings may only be generalizable to patients with commercial insurance.

## Background

Major depressive disorder (MDD) is one of the most common mental health disorders in the United States. In 2017, the National Survey on Drug Use and Health estimated that 17.3 million adults in the United States—representing 7.1% of the adult population—had experienced at least one major depressive episode during the previous year [1]. The economic costs of MDD are substantial. In fact, the incremental economic burden of adults with MDD in the United States was estimated at $210.5 billion in 2010, of which 47% ($98.8 billion) resulted from direct medical and pharmaceutical costs associated with MDD, and 53% ($111.7 billion) resulted from indirect costs [2]. Indirect costs included costs related to suicide ($9.7 billion) and costs resulting from the impact of MDD in the workplace ($102 billion).

Treatments for MDD include pharmacotherapy, psychotherapy, and somatic therapies, such as electroconvulsive therapy. The Practice Guideline from the American Psychiatric Association recommends that patients who respond to initial treatment with antidepressants in the acute phase should continue to receive pharmacotherapy for 4 to 9 months to reduce the risk of relapse [3]. Traditionally, a relapse of MDD is defined as the reemergence of MDD symptoms following remission of those symptoms but preceding recovery, while a recurrence is the onset of a new episode of depression following recovery [4].

However, evidence suggests that 30% to 85% of patients experience a relapse or recurrence of MDD [5, 6]. Patients who experience relapse or recurrence face an increased risk of another relapse or recurrence. Data indicate that the risk of a subsequent recurrence increases by 16% after each recurrence [7]. Risk factors for recurrence include the presence of residual symptoms despite antidepressant therapy, a history of previous recurrence, a history of or current comorbid anxiety disorder, and a history of child maltreatment or abuse [8].

Given the physical, psychosocial, and economic impact of MDD episodes, preventing relapses of depression is a critical component of managing MDD. Although antidepressants effectively reduce the risk of relapse [6], relapse rates are higher in patients on antidepressants who are not adherent or who discontinue treatment early than in those who use antidepressants as directed [9]. Evidence suggests that the median time to recurrence in patients continuing antidepressant therapy is substantially longer than for patients who prematurely discontinue therapy [6]. In one study, the median time to recurrence was approximately 40 months in patients continuing therapy, compared with a little over 1 year in patients who discontinued antidepressant therapy [6]. Moreover, patients who are nonadherent to medication are more likely to experience increased risks of relapse or recurrence, emergency department (ED) visits, and hospitalization [9].

One factor that can affect patient adherence to antidepressant therapy (generally defined as the extent to which patients use medication as directed) and persistence (defined as the duration of time until patients discontinue medication) is the type of antidepressant used [10]. Adherence to and persistence with antidepressant medication are generally poor. Results from a retrospective claims analysis of insured patients in the United States reported that by 3 months, only 42% to 47% were at least 80% adherent to therapy with selective-serotonin reuptake inhibitors (SSRIs) and serotonin and norepinephrine reuptake inhibitors (SNRIs) and 24% were adherent to therapy with tricyclic antidepressants (TCAs). By 12 months, the percentages of patients who were at least 80% adherent were 21% to 26% with SSRIs/SNRIs and 11% with TCAs. Rates of persistence were even lower. At 1 year, only 17% were persistent with SSRI therapy, 22% with SNRI therapy, and 9% with TCAs [10].

Given the low rates of adherence and persistence and the high rates of relapse among patients with MDD, the clinical and economic burden of relapse in MDD represents a significant concern, and additional data are needed to better quantify its impact in the real world. Consequently, we conducted this retrospective real-world study of medical and pharmacy claims to assess the incremental health care burden of relapse in patients with MDD.

## Methods

### Study design

This real-world retrospective cohort study analyzed data from patients enrolled in commercial health plans using administrative medical and pharmacy claims data collected between January 1, 2011, and September 30, 2018 (defined as the study period). We used data from the medical/pharmacy database of Magellan Health, Inc. This database consists of claims data submitted by health plans that have contracted to receive various services from Magellan. These data were derived from populations across the continental United States participating in regional health plans and contain adjudicated paid claims that represent submissions by the providers. These data included information commonly required in institutional, professional, and pharmacy claims, such as dates of service, provider information, procedure codes, drug prescriptions, and financial information. Claims data were considered only after adjudication. Data were validated within tolerance limits. Quality checks were performed on the data, one health plan at a time, before they were included in the study. Monthly claim counts, patient counts, and allowed and paid amount totals were verified for consistency for this study.

### Study population

To be included in the study, patients were required to have a diagnosis of MDD (*International Classification of Diseases, Ninth Revision, Clinical Modification* [ICD-9-CM] codes 296.2x, 296.3x; and *Tenth Revision* [ICD-10-CM] codes F32.xx, F33.xx) on at least two separate claims and have at least one claim for an antidepressant indicated for depression in the identification period (between January 1, 2012, and September 30, 2017). Patients were also required to have a 6-month antidepressant-free period before the index date (defined as the first prescription for an antidepressant during the study period) to ensure that they were incident antidepressant users. Patients must also have been ≥18 years of age on the index date and continuously enrolled in both medical and pharmacy benefits for at least 12 months prior to the index date through at least 12 months after the index date. Patients who were pregnant or who had a diagnosis of schizophrenia or bipolar disorder were excluded from the study.

Patients who met the inclusion and exclusion criteria were divided into two cohorts: patients with relapse and patients without relapse. Patients were considered to experience a relapse if the database confirmed that they experienced a suicide attempt, psychiatric hospitalization, mental health−related ED visit, or use of electroconvulsive therapy, or if they reinitiated antidepressant medication after a gap of ≥6 months following the previous antidepressant prescription. This definition has been used previously in published methodology to define relapse in MDD using claims data [11].

Patients with relapse were matched using propensity scores to patients without relapse based on their age group, sex, region, health plan type, and select comorbidities. Comorbidities were selected after a review of the literature and advice from medical teams (from Takeda and Lundbeck) to utilize a wide range of the most common psychiatric and physical comorbidities; these comorbidities are shown in Table 1.

### Endpoints and statistical analysis

Key study assessments included annual all-cause and mental health–related health care resource utilization and costs during the baseline period (defined as the 12-month period prior to the index date) and annual all-cause and MDD–related utilization and costs during the follow-up period (defined as the ≥12-month period after the index date), as well as evaluations of adherence and persistence with the index medication. Adherence to the index medication was assessed by evaluating the proportion of days covered (PDC), calculated as the number of days covered by supply of medication in a specific period divided by the number of days in that period, and the medication possession ratio (MPR), calculated as the sum of days’ supply for all fills in a specific period divided by the number of days in that period. The proportion of adherent patients was also evaluated by calculating the percentage of patients with a PDC ≥0.80 and, in a separate analysis, an MPR ≥0.80. Finally, treatment persistence, defined as the number of days patients continue using their index antidepressant, was also assessed. Persistence was defined as the number of days from the index date to the earliest of the following: the ending date of the last prescription, the date of the first gap of more than 30 days between prescriptions, or the end of the study period. Therefore, patients were considered to be persistent with their index antidepressant if they did not have a gap in their index antidepressant treatment of > 30 days [12].

Statistical differences between patients with relapse and without relapse were assessed using the chi-square or Fisher's exact test, as appropriate (for categorical variables), or *t* tests (for continuous variables). Statistical tests were 2-sided with a significance threshold of *p* < .05. All costs were adjusted to 2018 US dollars using the change in the medical component of the Consumer Price Index.

## Results

### Patient disposition and demographics

**Fig. 1:**
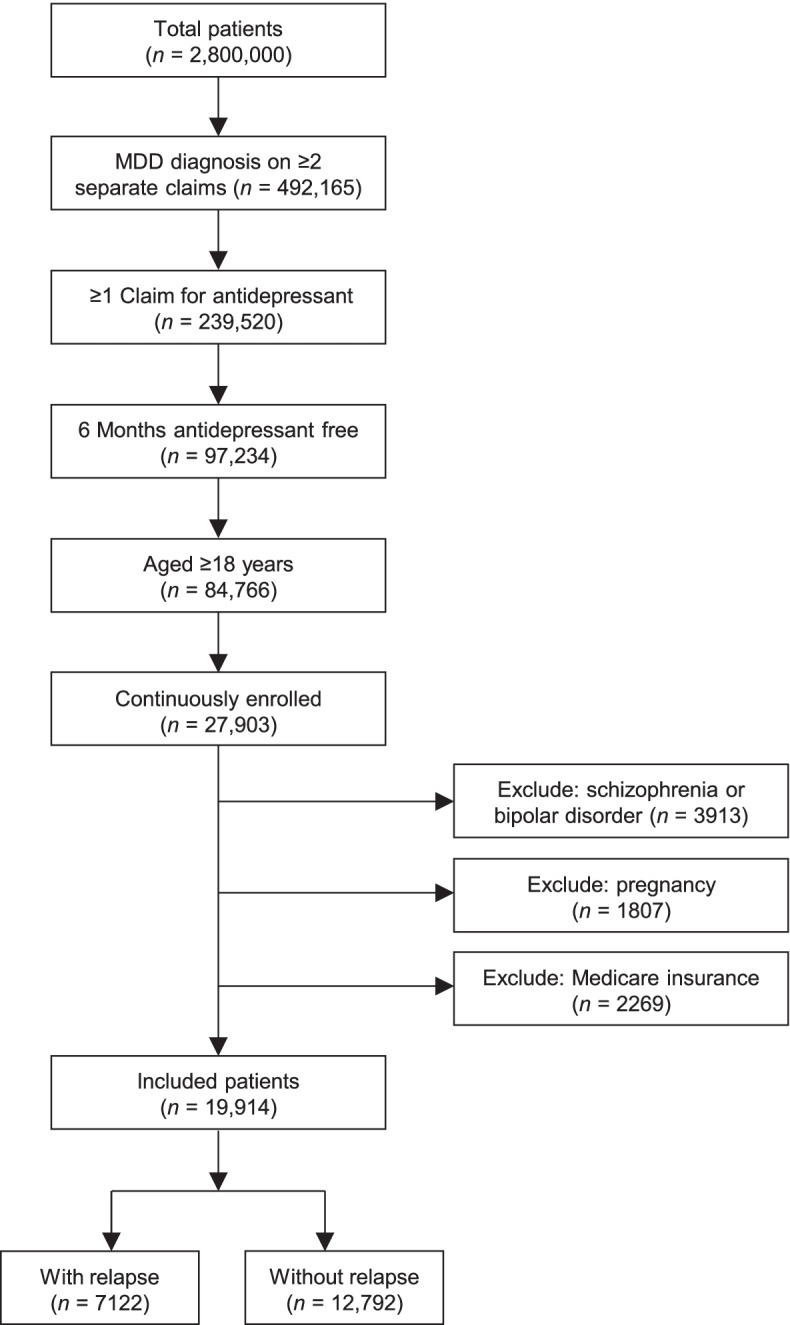
Patient Attrition. Patient disposition and attrition. *MDD,* major depressive disorder

Medical and pharmacy claims data were available during the study period from a total of 2.8 million patients (Fig. 1). Of these, 492,165 (3.9%) had a diagnosis of MDD on at least two separate claims on distinct dates of service. Application of the remaining eligibility criteria reduced the population to 19,914 patients, of whom 7122 were patients with relapse and 12,792 were patients who did not relapse (Fig. 1).

Most patients with relapse (81.2%) were identified using the criterion “reinitiation of treatment after a gap of ≥6 months following previous antidepressant prescription.” Matching the patients with relapse to those without relapse using propensity scores resulted in a final study population of 7093 patients with relapse and 7093 matched patients without relapse. These two sub-cohorts of matched patients had similar demographic characteristics and similar occurrence of comorbid conditions (Table 1).

**Table 1:** Patient Characteristics and Comorbidities Before and After Matching (Demographics)

	Before matching			After matching		
	Patients with relapse (***n*** = 7122)	Patients without relapse (***n*** = 12,792)	***p*** value	Patients with relapse (***n*** = 7093)	Patients without relapse (***n*** = 7093)	***p*** value
**Characteristics**						
Age, years						
Mean (SD)	42.0 (13.4)	42.4 (13.0)	.0482	42.0 (13.4)	41.9 (13.3)	.7736
Age group, years, *n* (%)						
18 − 29	1601 (22.5)	2586 (20.2)	.0053	1591 (22.4)	1589 (22.4)	.9789
30 − 39	1223 (17.2)	2336 (18.3)		1222 (17.2)	1226 (17.3)	
40 − 49	1796 (25.2)	3313 (25.9)		1791 (25.3)	1811 (25.5)	
50 − 59	1871 (26.3)	3444 (26.9)		1867 (26.3)	1873 (26.4)	
60 − 69	612 (8.6)	1082 (8.5)		606 (8.5)	578 (8.1)	
70 − 79	15 (0.2)	29 (0.2)		15 (0.2)	14 (0.2)	
≥ 80	4 (0.1)	2 (0.0)		1 (0.0)	2 (0.0)	
Sex, *n* (%)						
Female	4546 (63.8)	7926 (62.0)	.0090	4524 (63.8)	4539 (64.0)	.8067
Male	2576 (36.2)	4866 (38.0)		2569 (36.2)	2554 (36.0)	
Plan type, *n* (%)^a^						
PPO/POS	4132 (58.0)	7767 (60.7)	<.0001	4123 (58.1)	4088 (57.6)	.6854
HMO	883 (12.4)	1728 (13.5)		883 (12.4)	916 (12.9)	
Other	2107 (29.6)	3297 (25.8)		2087 (29.4)	2089 (29.5)	
**Comorbidities**						
Substance/alcohol abuse disorder	1409 (19.8)	1677 (13.1)	<.0001	1383 (19.5)	1399 (19.7)	.7511
Type 2 diabetes mellitus	740 (10.4)	1224 (9.6)	.0630	731 (10.3)	675 (9.5)	.1222
Obesity	1047 (14.7)	1886 (14.7)	.9501	1043 (14.7)	971 (13.7)	.0876
Hyperlipidemia	2282 (32.0)	4190 (32.8)	.3050	2267 (32.0)	2175 (30.7)	.0995
Hypertension	2021 (28.4)	3497 (27.3)	.1167	2003 (28.2)	1857 (26.2)	.0062
Chronic kidney disease	100 (1.4)	164 (1.3)	.4775	97 (1.4)	90 (1.3)	.6588
Coronary heart disease	297 (4.2)	540 (4.2)	.8829	292 (4.1)	248 (3.5)	.0591
Congestive heart failure	202 (2.8)	396 (3.1)	.3192	201 (2.8)	161 (2.3)	.0377
Cerebrovascular disease	317 (4.5)	434 (3.4)	.0002	304 (4.3)	285 (4.0)	.4487
Peripheral vascular disease	245 (3.4)	382 (3.0)	.0826	240 (3.4)	230 (3.2)	.6729
Cancer	2296 (32.2)	4082 (31.9)	.6346	2280 (32.1)	2254 (31.8)	.6526
Anxiety disorder	3749 (52.6)	6111 (47.8)	<.0001	3722 (52.5)	3653 (51.5)	.2531
Sleeping disorder	1374 (19.3)	2222 (17.4)	.0008	1360 (19.2)	1313 (18.5)	.3233
COPD	291 (4.1)	368 (2.9)	<.0001	277 (3.9)	265 (3.7)	.6300
Parkinson disease	15 (0.2)	19 (0.1)	.3705	14 (0.2)	14 (0.2)	>.99
Multiple sclerosis	43 (0.6)	79 (0.6)	>.99	43 (0.6)	39 (0.5)	.7399
Gastrointestinal disorder	1590 (22.3)	2582 (20.2)	.0004	1573 (22.2)	1591 (22.4)	.7317

*COPD* Chronic obstructive pulmonary disease, *HMO* Health maintenance organization, *POS* Point of service, *PPO* Preferred provider organization, *SD* Standard deviation

^a^PPO/POS insurance plans do not require specialist referrals; they cover out-of-network care, but at a higher cost to the patient compared with in-network care. HMO plans require referral from a primary care physician for patients to see specialist providers; also, HMOs typically do not cover services outside the plan’s provider network. “Other” plans included anything other than HMO or PPO/POS plans and were typically custom-designed programs for large employers that resemble PPO/POS plans

### Health care resource utilization and costs during the baseline period

**Fig. 2:**
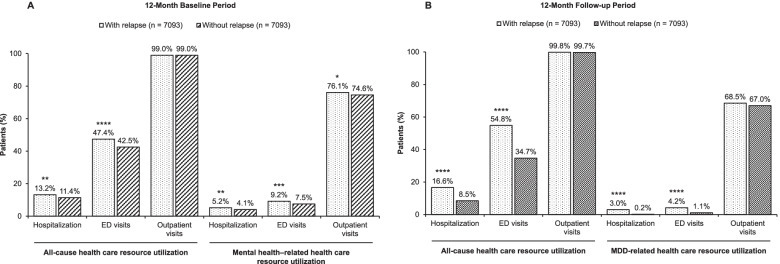
Proportion of Patients Utilizing Health Care Resources During 12-Month Baseline (**A**) and 12-Month Follow-up (**B**) Periods. **A** For the baseline period, mental health–related health care resource utilization is reported, whereas (**B**) for the follow-up period, MDD-related health care resource utilization is reported. *ED,* emergency department; *MDD,* major depressive disorder. *p < .05; **p < .01; ***p < .001; ****p < .0001 vs patients without relapse

A statistically significant association was found between occurrence of relapse and occurrence of hospitalization or ED visits for any cause and for mental health–related causes during the baseline period (Fig. 2A; Table 2). Annual costs during the baseline period were similar for patients with relapse and patients without relapse (Table 2).

**Table 2:** Annual All-Cause and Mental Health–Related Health Care Resource Utilization and Costs During the Baseline Period

	Annual all-cause health care resource utilization and costs			Annual mental health–related health care resource utilization and costs		
	Patients with relapse (***n*** = 7093)	Patients without relapse (***n*** = 7093)	***p*** value	Patients with relapse (***n*** = 7093)	Patients without relapse (***n*** = 7093)	***p*** value
Utilization, *n* (%)						
Hospitalizations	933 (13.2)	810 (11.4)	.0018	372 (5.2)	294 (4.1)	*.0022*
ED visits	3365 (47.4)	3014 (42.5)	*<.0001*	652 (9.2)	533 (7.5)	*.0003*
Outpatient visits	7024 (99.0)	7023 (99.0)	>.99	5397 (76.1)	5289 (74.6)	*.0372*
Costs in US$, mean (SD)						
Medical costs	5392 (11,056)	5513 (15,904)	.5978	682 (1971)	632 (1512)	.0894
Pharmacy costs	1278 (4307)	1333 (5324)	.4952	82 (609)	97 (1434)	.4289
Total costs (pharmacy and medical)	6669 (12,471)	6846 (17,726)	.4921	765 (2079)	729 (2103)	.3124

Significant *p* values (< .05) are shown in italics

*ED* Emergency department, *SD* Standard deviation

### Health care resource utilization and costs during follow-up

During the follow-up period, patients with relapse had significantly higher rates of all-cause hospitalization (16.6% vs 8.5%; *p* < .0001) and all-cause ED visits (54.8% vs 34.7%; *p* < .0001) than patients without relapse (Fig. 2B; Table 3). Rates of MDD-related hospitalization and ED visits were also significantly higher in patients with relapse than in those without (Table 3).

**Table 3:** All-Cause and MDD-Related Health Care Resource Utilization During the Follow-up Period

	All-cause health care resource utilization			MDD-related health care resource utilization		
	Patients with relapse (***n*** = 7093)	Patients without relapse (***n*** = 7093)	***p*** value	Patients with relapse (***n*** = 7093)	Patients without relapse (***n*** = 7093)	***p*** value
Average follow-up time, mean (SD), months	27.53 (10.93)	25.99 (9.61)	*<.0001*	27.53 (10.93)	25.99 (9.61)	*<.0001*
Hospitalizations, *n* (%)	1175 (16.6)	603 (8.5)	*<.0001*	214 (3.0)	15 (0.2)	*<.0001*
ED visits, *n* (%)	3890 (54.8)	2463 (34.7)	*<.0001*	298 (4.2)	76 (1.1)	*<.0001*
Outpatient visits, *n* (%)	7078 (99.8)	7070 (99.7)	.2552	4857 (68.5)	4755 (67.0)	.0696
Per-patient data, mean (SD)						
Hospitalizations	0.16 (0.59)	0.07 (0.37)	*<.0001*	0.02 (0.14)	0.00 (0.05)	*<.0001*
Length of stay, days	0.83 (4.24)	0.43 (3.61)	*<.0001*	0.09 (0.69)	0.01 (0.36)	*<.0001*
ED visits	0.97 (2.76)	0.55 (1.96)	*<.0001*	0.06 (0.71)	0.05 (1.05)	.5434
Laboratory visits	0.04 (0.27)	0.03 (0.19)	*.0062*	0.04 (0.27)	0.03 (0.19)	*.0062*
Outpatient visits	19.65 (17.69)	17.45 (16.69)	*<.0001*	4.44 (8.66)	3.94 (7.96)	*.0004*
Primary care visits	3.29 (3.43)	2.92 (3.13)	*<.0001*	0.19 (0.61)	0.19 (0.67)	.7074
Psychiatrist visits	2.21 (4.66)	1.82 (4.12)	*<.0001*	1.52 (3.91)	1.33 (3.58)	*.0017*
Behavioral therapy visits	5.98 (11.07)	5.55 (10.88)	*.0196*	3.14 (7.86)	2.90 (7.52)	.0634
Prescriptions	7.23 (5.75)	6.03 (5.14)	*<.0001*	1.60 (1.14)	1.27 (1.09)	*<.0001*

Significant *p* values (<.05) are shown in italics

*ED* Emergency department*, MDD* Major depressive disorder, *SD* Standard deviation

A statistically significant association was also observed between relapse and higher costs. The mean total annual all-cause cost was $2149 greater for patients with relapse than for matched patients without relapse ($12,594 vs $10,445; *p* < .0001; Table 4). In addition, mean total MDD-related cost was significantly greater for patients with relapse than for matched patients without relapse ($1038 vs $863; *p* < .0001; Table 4).

**Table 4:** Annual all-cause and MDD-related costs during the follow-up period

	Annual all-cause costs (US$)			Annual MDD-related costs (US$)		
	Patients with relapse (***n*** = 7093)	Patients without relapse (***n*** = 7093)	***p*** value	Patients with relapse (***n*** = 7093)	Patients without relapse (***n*** = 7093)	***p*** value
Hospitalization costs	785 (4959)	573 (4713)	*.0089*	32 (469)	3 (46)	*<.0001*
ED costs	1028 (3451)	429 (1344)	*<.0001*	24 (222)	7 (158)	*<.0001*
Laboratory costs	558 (2023)	422 (1484)	*<.0001*	9 (85)	8 (91)	.3310
Outpatient costs	6391 (16,625)	5537 (14,548)	*.0011*	703 (1773)	591 (1498)	*<.0001*
Other costs	1242 (4930)	1122 (5349)	.1671	19 (213)	11 (217)	*.0284*
Medical costs	10,004 (21,126)	8084 (19,460)	*<.0001*	787 (1976)	619 (1553)	*<.0001*
Pharmacy costs	2590 (7593)	2361 (9827)	.1210	251 (893)	244 (722)	.5997
Total costs (pharmacy and medical)	12,594 (24,003)	10,445 (23,288)	*<.0001*	1038 (2201)	863 (1763)	*<.0001*

Significant *p* values (<.05) are shown in italics

*ED* Emergency department*, MDD* Major depressive disorder, *SD* Standard deviation

^a^Costs reported as US$ mean (SD)

### Adherence and persistence during follow-up

**Fig. 3:**
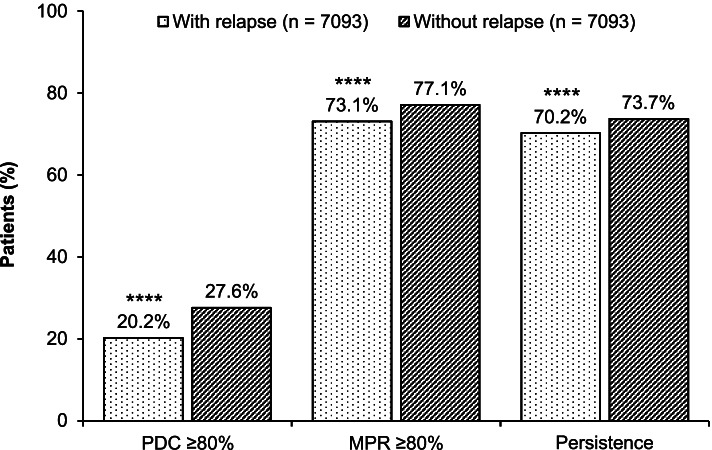
Adherence and Persistence to the Index Antidepressant During Follow-up. Patients who achieved medication persistence were defined as those who did not have a gap in their index antidepressant treatment exceeding 30 days during 12 months of follow-up. *MPR,* medication possession ratio; *PDC,* proportion of days covered. ****p < .0001 vs patients without relapse

The proportions of adherent patients (MPR or PDC ≥0.80) were significantly lower among patients with relapse compared with patients without relapse (Fig. 3). Using the PDC ≥0.80 criterion for adherence, the percentages of adherent patients were 20.2% in patients with relapse and 27.6% in patients without relapse (*p <* .0001). The mean PDC was 0.43 in patients with relapse vs 0.49 in patients without relapse (*p <* .0001).

Using the MPR ≥0.80 criterion for adherence, 73.1% of patients with relapse were considered adherent compared with 77.1% of patients without relapse (*p <* .0001). Mean MPR was 0.85 in patients with relapse vs 0.88 in patients without relapse (*p <* .0001).

The proportion of patients who were persistent with their index antidepressant at 1 year was also significantly lower in patients with relapse than in patients without relapse (70.2% vs 73.7%; *p <* .0001). The mean persistence was 135.1 days among patients with relapse and 159.2 days among those who did not relapse (*p <* .0001).

## Discussion

Our study demonstrated statistically significant associations between occurrence of relapse in patients with MDD and higher rates of hospitalization, visits to the ED, and outpatient visits. The significantly greater use of health care resources among patients who relapsed was accompanied by significantly higher total and MDD-related annual costs. Moreover, rates of adherence and persistence were significantly lower among patients who relapsed than among those who did not during the treatment period. The number of prescriptions per patient was higher among those who relapsed, which we see as part of an overall pattern of higher health care resource utilization and need for medical support during relapse.

Our findings are consistent with those of a recent retrospective analysis of 22,236 patients with MDD treated with a branded antidepressant medication who were selected from the Truven Health Analytics MarketScan Databases between 2004 and 2015 [13]. In that study, approximately 25% of patients treated with antidepressant medications had at least one indicator of MDD relapse or recurrence during the 3-year period after treatment initiation. Among patients with indicators of relapse or recurrence, health care resource utilization and costs were significantly higher than for patients without such signs ($20,590 vs $12,368; *p <* .001). Rates of ED visits, inpatient days, and inpatient admissions were more than two times higher after the relapse or recurrence than among patients without relapse or recurrence. The authors noted that patients with signs of relapse and recurrence presented with a more complex profile of MDD, including a more extensive history of antidepressant treatment, greater comorbidities (including higher rates of anxiety disorders), and higher rates of inpatient admissions and ED visits at baseline, suggesting that these patients faced a higher risk of relapse and recurrence [13].

Given the impact of adherence and persistence on rates of relapse and the association between relapse and health care resource utilization and economic costs, interventions designed to facilitate adherence and persistence in patients with MDD at risk for relapse could ultimately improve outcomes and reduce costs. Additional research is needed to establish a causal connection between relapse and resource utilization and to identify which interventions are most likely to improve adherence and persistence, reduce the risk of relapse, and minimize costs.

### Limitations

Our study does have limitations. First, real-world claims data can be prone to outliers that skew health care costs data, resulting in higher or lower than expected mean values. Moreover, studies with large sample sizes can detect small differences between groups that are statistically significant but might not be clinically significant. Consequently, the clinical relevance of statistically significant differences should be evaluated when small statistically significant differences are observed in large studies.

Similar to other claims-based analyses, our data are dependent on professional ICD-9/10 coding. In the clinical setting, different professional types might have different coding patterns, and not all coding may be accurate. In addition, services performed but not billed are not captured. Reasons for patterns in the data are not always knowable; for example, discontinuation of treatment may in some cases be a result of prescribers’ decisions rather than lack of patient adherence. Similarly, socioeconomic background may play a role in the likelihood that a patient may relapse, but such data are not available within the claims database. We also note that the comorbid conditions selected for propensity score matching did not include thyroid disease or comorbid personality disorders; this may be considered as a limitation in our analysis, as these conditions may impact MDD relapse. Finally, given that our study only includes patients in the United States who are commercially insured, our results might not be generalizable to patients insured by noncommercial plans, such as Medicaid, or to health care systems in other countries.

## Conclusions

Our findings demonstrate that MDD relapses are associated with an increase in total health care costs and health care utilization, as well as lower rates of adherence to and persistence with MDD medication. Future research is needed to determine whether there is a causal link between MDD relapse and the level of health care utilization. Findings from such research may help determine whether programs that encourage adherence to antidepressant medications after a diagnosis of MDD would potentially reduce relapse rates and, therefore, reduce costs and the health care burden related to relapse.

## Data Availability

The authors confirm that the data supporting the findings of this study are available within the article. The corresponding author may be contacted for further data sharing: Maëlys Touya, mtou@lundbeck.com.

## Abbreviations

ED: Emergency department
ICD-9-CM: International Classification of Diseases, Ninth Revision, Clinical Modification
ICD-10-CM: International Classification of Diseases, Tenth Revision, Clinical Modification
MDD: Major depressive disorder
MPR: Medication possession ratio
PDC: Proportion of days covered
SNRI: Serotonin and norepinephrine reuptake inhibitor
SSRI: Selective serotonin reuptake inhibitor
TCA: Tricyclic antidepressant
